# Identification of Potentially Relevant Genes for Excessive Exercise-Induced Pathological Cardiac Hypertrophy in Zebrafish

**DOI:** 10.3389/fphys.2020.565307

**Published:** 2020-11-30

**Authors:** Zuoqiong Zhou, Lan Zheng, Changfa Tang, Zhanglin Chen, Runkang Zhu, Xiyang Peng, Xiushan Wu, Ping Zhu

**Affiliations:** ^1^Guangdong Cardiovascular Institute, Guangdong Provincial People’s Hospital, Guangdong Academy of Medical Sciences, Guangzhou, China; ^2^Key Laboratory of Physical Fitness and Exercise Rehabilitation of Hunan Province, College of Physical Education, Hunan Normal University, Changsha, China

**Keywords:** pathological cardiac hypertrophy, RNA-seq, autophagy, FoxO signaling pathway, excessive exercise

## Abstract

Exercise-induced cardiac remodeling has aroused public concern for some time, as sudden cardiac death is known to occur in athletes; however, little is known about the underlying mechanism of exercise-induced cardiac injury. In the present study, we established an excessive exercise-induced pathologic cardiac hypertrophy model in zebrafish with increased myocardial fibrosis, myofibril disassembly, mitochondrial degradation, upregulated expression of the pathological hypertrophy marker genes in the heart, contractile impairment, and cardiopulmonary function impairment. High-throughput RNA-seq analysis revealed that the differentially expressed genes were enriched in the regulation of autophagy, protein folding, and degradation, myofibril development, angiogenesis, metabolic reprogramming, and insulin and FoxO signaling pathways. FOXO proteins may be the core mediator of the regulatory network needed to promote the pathological response. Further, PPI network analysis showed that *pik3c3*, *gapdh*, *fbox32*, *fzr1*, *ubox5*, *lmo7a*, *kctd7*, *fbxo9*, *lonrf1l*, *fbxl4*, *nhpb2l1b*, *nhp2*, *fbl*, *hsp90aa1.1*, *snrpd3l*, *dhx15*, *mrto4*, *ruvbl1*, *hspa8b*, and *faub* are the hub genes that correlate with the pathogenesis of pathological cardiac hypertrophy. The underlying regulatory pathways and cardiac pressure-responsive molecules identified in the present study will provide valuable insights for the supervision and clinical treatment of pathological cardiac hypertrophy induced by excessive exercise.

## Introduction

The most frequent medical cause of death in athletes is sudden cardiac death (SCD), which usually occurs during intensive training (Harmon et al., 2014). Despite the widely recognized benefits of regular exercise and physical activity, excessive exercise may lead to increased risk of arrhythmia, myocardial ischemia, myocardial fibrosis, and even sudden cardiac death (Siscovick et al., 1984; La Gerche et al., 2012; Galderisi et al., 2015). Given the increasing extensive development of mass sports, uncovering the pathogenic mechanism of exercise-related cardiac disease is urgently required for the prevention of cardiac injury and death.

Moderate exercise regimes can induce benign physiological cardiac hypertrophy, resulting in increased ventricular stroke volume and cardiac output (Pelliccia et al., 1991, 1999). There is strong evidence that exercise is related to a reduced risk of cardiovascular disease, brought about by counteracting structural and functional pathological cardiac changes (Garciarena et al., 2009; Oliveira et al., 2009; Melo et al., 2014; Ooi et al., 2014). However, long-term intense exercise leads to pathological cardiac hypotrophy and heart failure. An increase in cardiomyocyte apoptosis, necrosis, autophagic cell death, and fibrosis occur after long-term excessive training, which could induce the shift from physiological cardiac hypertrophy to pathological cardiac hypotrophy (Carraro and Franceschi, 1997; Nakai et al., 2007; Oka and Komuro, 2008). The focus of research to date has been on cellular and molecular mechanisms of physiological cardiac hypertrophy induced by exercise due to its cardioprotective effects (Ooi et al., 2014). However, the protective and destructive mechanisms induced by exercise are at the root of the development of pathogenic cardiac hypotrophy. In overtraining, an imbalance between cell damage and immune system response leads to pathological remodeling of the heart and, eventually, to heart failure (Schultz et al., 2007). Some of the physiological activities of cardiomyocytes, including cell fibrosis, protein synthesis, mitochondrial dysfunction, metabolic reprogramming, and angiogenesis, have been implicated in pathological hypertrophy, but the specific molecular mechanism and molecular regulatory network of exercise-induced pathogenic cardiac hypotrophy remains unclear. Understanding this relationship will enable effective prevention and control of exercise overload and heart injury, and improve the medical supervision of athletes.

Zebrafish has become a popular vertebrate model to study the pathogenesis of human diseases, such as cardiomyopathy, because of its convenience and amenability for genetic manipulation, genetic proximity, and relatively small genome (Bournele and Beis, 2016; Dvornikov et al., 2018). To gain understanding of the mechanisms by which excessive exercise exerts adverse cardiac effects, we constructed the first zebrafish overtraining model with the phenotype of pathological cardiac hypotrophy, and applied RNA-seq to systematically identify the key genes and signaling pathways involved in adverse cardiac remodeling in response to excessive exercise.

## Materials and Methods

### Zebrafish Husbandry and Swimming Training

AB strain zebrafish were raised under 14 h of light at 28°C under standard husbandry conditions. Sixty male zebrafish with the same parent, same age, and similar body length and weight were randomly divided into two groups. Before training, the critical swimming velocity of zebrafish (Ucrit) was tested according to a previously reported protocol using Loligo® Systems (#SW10600) (Palstra et al., 2010). Zebrafish were subjected to exercise in a current of 24 cm/s, which corresponds to 80% of their initial Ucrit, for 4 weeks (6 h for 6 days per week). The control group zebrafish remained in housing tanks for 4 weeks.

### Transmission Electron Microscopy

Zebrafish heart samples were dissected and fixed with 2.5% glutaraldehyde in 0.1 M/L phosphate buffer for 12 h. The fixation buffer was discarded and samples were washed with phosphate buffered saline (PBS) for 1 h and then fixed with 1% osmium tetroxide at 4°C for 2 h. Samples were dehydrated in a series of ethanol concentrations, embedded in Eponate 12 Resin (Ted Pella, United States), and processed into ultrathin sections. The ultrathin sections were stained with uranyl acetate and lead citrate and examined under a Hitachi H7700 transmission electron microscope.

### Masson Staining

Heart samples from each group were harvested, fixed in 4% paraformaldehyde, embedded in paraffin, and sectioned. The sections were deparaffinized, rehydrated through a series of alcohol concentrations, and washed in distilled water. The sections were then stained in Biebrich scarlet-acid fuchsin solution and washed in distilled water again. The sections were differentiated in phosphomolybdic–phosphotungstic acid solution, stained with aniline blue solution, and rinsed in distilled water. The sections were differentiated in 1% acetic acid solution, washed in distilled water, dehydrated in a series of ethyl alcohol concentrations, cleared in xylene, and mounted with resinous mounting medium for microscopic analysis.

### Measurement of the Rate of Consumption of Oxygen

Consumption of oxygen was also conducted in Loligo Systems with a mini swim tunnel respirometer, consisting of a fiber optic oxygen probe, DAQ-M automated oxygen measurement system, and AutoRespTM 1 software. Measurement of the rate of consumption of oxygen (MO_2_) was performed by automated intermittent-flow respirometry in loops of 7 min. Each loop consisted of a 5 min measuring phase followed by a 90 s flushing phase and 30 s waiting phase. Before the measurement, the zebrafish fasted for 24 h and swam at a speed of 0.8 bl/s for 2 h to eliminate the sense of stress. After this acclimation period, the velocity was steadily increased (2.7 bl/s every 20 min) until the fish were unable to continue swimming due to fatigue. Concentrations of oxygen (O_2_) were measured using a fiber optic oxygen dipping probe which was connected to a Fibox 3 mini-sensor oxygen meter (Precision Sensing GmbH, Regensburg, DE).

### Reverse Transcriptase Quantitative PCR (RT-qPCR)

Total RNA was isolated from tissue using TRIzol (Invitrogen, CA) according to the manufacturer’s protocol. To analyze transcriptional changes, cDNA was generated using Superscript III reverse transcriptase (Invitrogen, CA), and RT-qPCR was performed using SYBR Green PCR Master Mix (Takara, Dalian, China). Standard PCR conditions were used to measure transcript abundance for 35 cycles in an ABI7500 machine (ABI Biosystems, Columbia, MD). The delta-delta Ct method was used to calculate the relative abundance of the tested genes. Primers used for expression analysis are listed in Supplementary Table 1.

### Identification of Differentially Expressed Genes

RNA was extracted from the hearts of the training group and the control group zebrafish to construct a cDNA library, which was sequenced in paired mode on an Illumina HiSeq6000. Clean data were obtained from the raw data after discarding adapter sequences and low-quality sequences using Trimmomatic (Bolger et al., 2014). We used the DESeq2 package to identify the differentially expressed genes (DEGs) between the two groups. DEGs were selected when the *P*-value was less than 0.05.

### Gene Ontology (GO) and Kyoto Encyclopedia of Genes and Genomes (KEGG) Enrichment Analysis of DEGs

Gene enrichment analysis was performed on the DEGs using the DAVID online database to identify physiological changes in overloaded hearts. A *P* < 0.05 was considered to be statistically significant for enrichment. KEGG pathway analysis was then used to identify biochemical pathways that were enriched for the DEGs, with an adjusted *p*-value of less than 0.05.

### Clusters of Orthologous Groups (COGs) Analysis of DEGs

We used the bioMart package to obtain the protein sequences of the DEGs, which were used for COGs analysis on the EggNOG online tool^1^.

### Protein–Protein Interaction (PPI) Network Construction

The PPI network was predicted using the search tool for the retrieval of interacting genes (STRING)^2^ online database. Interactions with a combined score of > 0.4 were considered to be statistically significant. The bioinformatics platform Cytoscape^3^ was used to visualize the molecular interaction network.

### Statistical Analysis

Data were analyzed using R software, version 3.6.3 (R Core Team, 2020), to estimate the homogeneity of variance, and Student’s *t*-tests were performed to compare the variance between experimental groups. A *p* < 0.05 was considered to be statistically significant.

### Echocardiography

Zebrafish in each group were anaesthetized in 0.16 mg/mL tricaine solution for 2 min and placed with the ventral side up in a small sponge holder. Echocardiography was then performed using a VisualSonics Vevo 2100 System echocardiograph (VisualSonics, Toronto, CA, United States) as described previously (Fang et al., 2020).

## Results

### Excessive Exercise Leads to Pathologic Cardiac Hypertrophy

**FIGURE 1:**
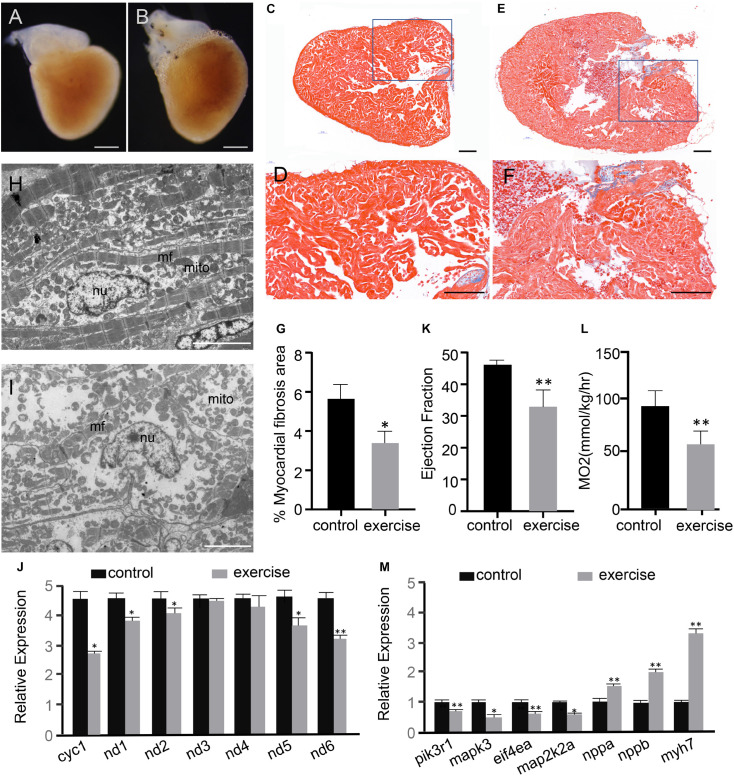
Excessive exercise leads to pathological cardiac hypertrophy. **(A,B)** Representative images from control **(A)** and excessively-exercised zebrafish hearts **(B)** after 4 weeks of static and excessive-exercise treatment. **(C**–**F)** Representative pictures of Masson’s trichrome-stained ventricular tissues of control **(C,D)** and excessively-exercised zebrafish hearts **(E,F)**. Blue area indicates fibrosis. **(D,F)** Are enlargements of the rectangle area in **(C,E)**, respectively. Scale bar = 100 μm. **(G)** Quantitative analysis of Masson staining positive myocardial fibrosis area using Image J. ^∗^*p* < 0.05 by unpaired Student’s *t*-test. *n* = 3. **(H,I)** TEM images of control **(H)** and excessively exercised heart tissue **(I)**. Scale bar = 5 μm. **(J)** RT-qPCR analysis of the expression of mitochondrial functional markers. ^∗^*p* < 0.05, ^∗∗^*p* < 0.01 by unpaired Student’s *t*-test. **(K)** Echocardiographic analysis of zebrafish after excessive exercise and in the static control (LVPW, LV posterior wall thickness). Error bars indicate SEM. *n* = 4. **(L)** The maximal oxygen consumption (MO_2_) of zebrafish after excessive exercise and in the control group was analyzed. *n* = 8. **(M)** RT-qPCR analysis of the expression of pathologic and physiological hypotrophy-related marker genes. ^∗^*p* < 0.05, ^∗∗^*p* < 0.01 by unpaired Student’s *t*-test.

After 4 weeks of excessive exercise, zebrafish hearts were dissected for microscopic observation. The hearts of zebrafish that were subjected to excessive exercise were evidently enlarged compared to those of the control zebrafish (Figures 1A,B and Supplementary Figure S1). Masson’s trichrome staining was performed to detect the accumulation of collagen fibrosis in the heart. According to the observed bright blue staining, collagen fibrosis was accumulating in both the interstitial and perivascular regions of the excessively exercised group (Figures 1E,F), while trichrome staining in the control zebrafish hearts was marginal (Figures 1C,D). The percentage of myocardial fibrosis area in the excessively exercised group was significantly larger than the control group (Figure 1G). The ultrastructure of the heart was analyzed using a transmission electron microscope. The muscle fibers and mitochondria were neatly arranged in the cardiomyocytes of the control group, while the muscle fibers and mitochondria of the excessively exercised group were arranged in a disorderly manner, and mitochondrial structural abnormalities and autophagy were observed (Figures 1H,I). Further detection of mitochondrial functional markers showed that mitochondrial cytochrome C protein Cyc1, NADH dehydrogenase 1 (nd1), NADH dehydrogenase 2 (nd2), NADH dehydrogenase 5 (nd5), and NADH dehydrogenase 6 (nd6) were significantly downregulated (Figure 1J). Echocardiographic analysis showed that zebrafish with excessive exercise-induced pathologic cardiac hypotrophy developed significant contractile impairment compared to the control group (Figure 1K and Supplementary Movies 1, 2). Oxygen consumption was used as an indicator of cardiopulmonary function. The maximal oxygen consumption of zebrafish after excessive exercise decreased significantly compared to in the control group (Figure 1L). RT-qPCR analysis showed that the embryonic genes *nppa*, *nppb*, and *myh7* showed elevated expression levels, while the members of the classic physiological signaling pathway *pik3r1*/PI3K, *mapk3*/ERK1, *eif4a*/eif4ea, and *map2k2a*/MEK2 were downregulated in the hearts of excessively exercised zebrafish compared to in the control group (Figure 1M). Taken together, these changes in the cardiac tissue suggest that excessive exercise can lead to pathologic cardiac hypertrophy.

### Differential Expression of Genes Caused by Excessive Exercise

**FIGURE 2:**
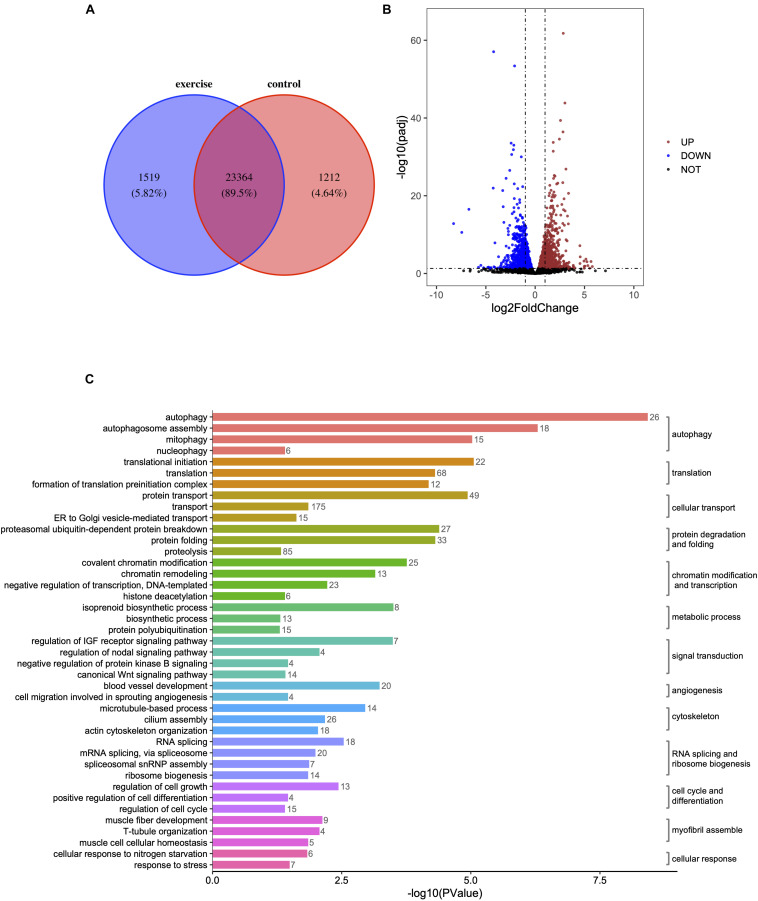
Identification of differentially expressed genes (DEGs) and enrichment analysis of GO terms in zebrafish hearts after excessive exercise and in the control group. **(A)** Venn diagram of the total number of identified genes in the excessive exercise and control groups. **(B)** Volcano plot constructed using fold-change values and *P* adj-values. Red: upregulated, Blue: downregulated. **(C)** The DEG enrichment analysis of GO terms in the biological process subcategory. The number indicates the number of DEGs that are enriched in the clustered representative terms.

High-throughput transcriptome analysis was used to unravel the underlying mechanisms of pathologic cardiac hypertrophy. We found that 1,519 genes (5.82%) were specifically expressed and 1,212 genes (4.64%) were absent in zebrafish hearts with pathological hypotrophy (Figure 2A) compared to in the control group. A total of 3,082 mRNAs were differentially expressed with a *p* < 0.05 in paired control and pathologic hypertrophy heart samples, of which 1,569 mRNAs were upregulated and 1,513 were downregulated (Figure 2B).

### Functional Annotation of DEGs

Gene ontology enrichment analysis was performed to investigate the biological functions of all DEGs. Autophagy, autophagosome assembly, mitophagy, translational initiation, protein transport, regulation of translational initiation, proteasomal ubiquitin-dependent protein breakdown, protein folding, translation, formation of translation preinitiation complex, and covalent chromatin modification were the top 10 enriched GO terms in the “biological processes” subcategory (Supplementary Table 2). Under the “molecular function” subcategory, the significantly enriched terms (*p* < 0.05) were associated with autophagy, transcription, cellular transport, protein folding and degradation, chromatin modification, and transcription, signal transduction, angiogenesis, cytoskeleton, RNA splicing, and ribosome biogenesis, cell cycle, and differentiation, myofibril assembly, metabolic process, and cellular response (Figure 2C).

**FIGURE 3:**
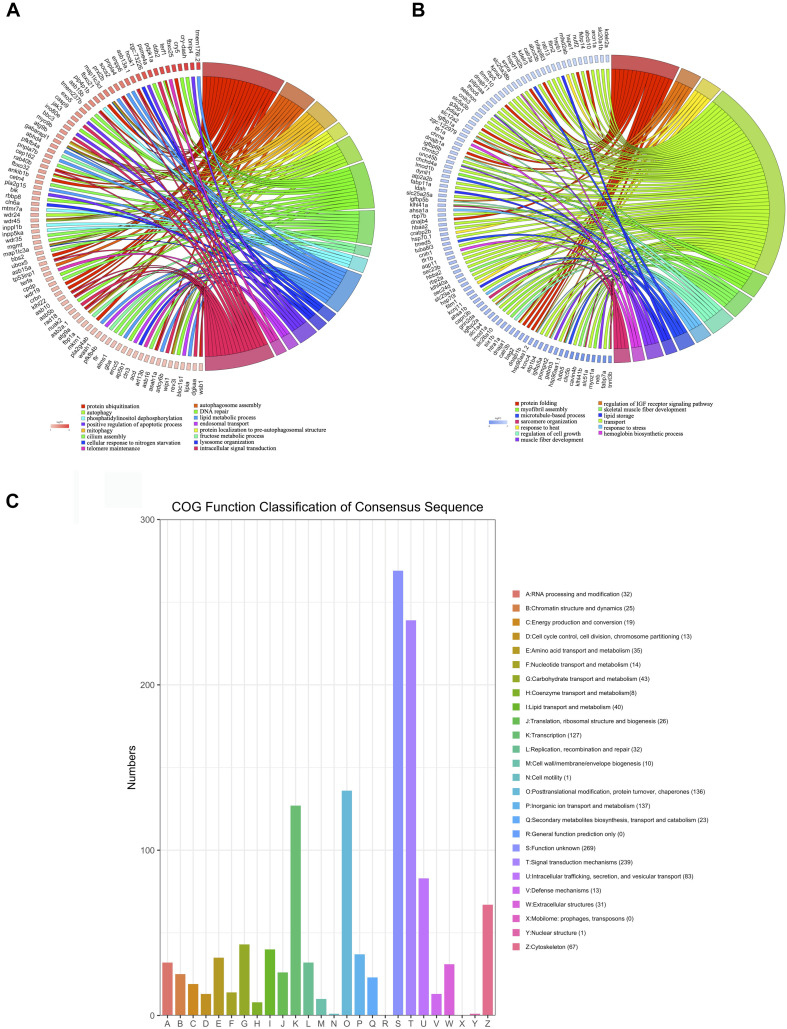
Function analysis of DEGs (*p* < 0.05, fold change > 2). **(A)** Upregulated DEGs were assigned to representative biological processes. **(B)** Downregulated DEGs were assigned to representative biological processes. **(C)** Cluster of Orthologous Groups (COG) functional classification of the DEGs. The legend shows the name of each function and the proportion of DEGs in each functional class.

To improve the specificity of the GO enrichment analysis, this process was repeated for genes that were up- or downregulated by at least twofold in the hearts of zebrafish exposed to excessive exercise. The upregulated genes were enriched in pathways related to autophagosome assembly, mitophagy, protein ubiquitination, and lipid, and fructose metabolic process. The downregulated genes were significantly enriched in response to heat, protein folding, sarcomere organization, regulation of insulin like growth factor receptor signaling pathway, and myofibril assembly (Figures 3A,B). We classified the DEGs (*p* < 0.05, fold change > 2) using the COG database, and identified that signal transduction mechanisms, posttranslational modification, protein turnover, chaperons, and transcription were the most representative functional clusters (Figure 3C).

**FIGURE 4:**
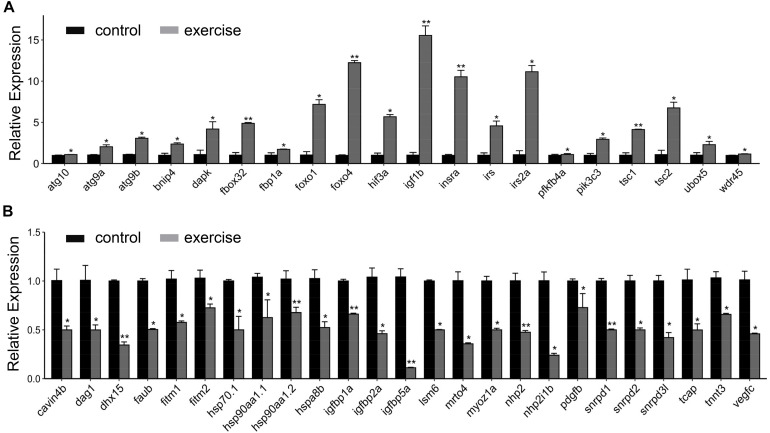
RT-qPCR validation of key genes involved in excessive exercise induced pathological cardiac hypertrophy. **(A)** upregulated genes involved in pathological remodeling. **(B)** downregulated genes involved in pathological remodeling. *n* = 8 in control and exercise group. *p < 0.05, **p < 0.01 by unpaired Student’s *t*-test.

Next, we aimed to identify the high-level GO terms that lead to pathological hypotrophy. We validated the expression of genes enriched in these GO terms and found that the expression of genes involved in autophagy (*atg101*, *agt9a*, *atg9b*, *tsc1*, and *tsc2*), FoxO signaling pathway (*foxo1*, *foxo4*, and *fbox32*), insulin/insulin-like signaling (*insra*, *igf1rb*, *irs*, and *irs2a*), glycolysis (*fbp1a* and *pfkfb4a*), and angiogenesis inhibitor hif3a were up-regulated (Figure 4A). Genes involved in protein folding (*hsp90aa1.1*, *hsp90aa1.2*, *hsp70.1*, and *hspad8b*), myofibril assembly (cavin4b, dag1, tcap3, and tnnt3), angiogenesis (*vegfc*, *pdgfbr*, and *hif1a*), and fatty acid storage (*fitm1* and *fitm2*) were down-regulated (Figure 4B).

### KEGG Pathway Analysis of DEGs

We mapped the DEGs to biological pathways using the KEGG pathway database. Most of the upregulated genes were enriched in lysosome, insulin resistance, regulation of autophagy, insulin signaling pathway, FoxO signaling pathway, mTOR signaling pathway, other glycan degradation, nucleotide excision repair, fructose, and mannose metabolism. Most of the downregulated genes were enriched in protein processing in the endoplasmic reticulum, spliceosome, proteasome, terpenoid backbone biosynthesis, focal adhesion, protein export, RNA transport, ribosome biogenesis in eukaryotes, phagosome, and tight junction (Table 1 and Supplementary Table 3).

**TABLE 1:** Enriched KEGG pathways in the heart tissue of excessively exercised zebrafish.

	ID	Term	Count	*P*-value
Up	dre04142	Lysosome	26	3.27E−06
	dre04931	Insulin resistance	20	6.97E−04
	dre04140	Regulation of autophagy	8	9.27E−04
	dre04910	Insulin signaling pathway	21	6.23E−03
	dre04068	FoxO signaling pathway	20	1.14E−02
	dre04150	mTOR signaling pathway	11	1.52E−02
	dre00511	Other glycan degradation	6	1.60E−02
	dre03420	Nucleotide excision repair	8	2.01E−02
	dre00051	Fructose and mannose metabolism	7	4.38E−02
Down	dre04141	Protein processing in endoplasmic reticulum	51	6.99E−12
	dre03040	Spliceosome	33	1.53E−06
	dre03050	Proteasome	18	1.44E−05
	dre00900	Terpenoid backbone biosynthesis	10	1.42E−04
	dre04510:	Focal adhesion	44	1.89E−04
	dre03060:	Protein export	8	4.32E−03
	dre03013	RNA transport	27	6.52E−03
	dre03008	Ribosome biogenesis in eukaryotes	15	1.53E−02
	dre04145	Phagosome	24	3.18E−02
	dre04530	Tight junction	18	4.19E−02

### Construction of PPI Network and Cluster Identification

**FIGURE 5:**
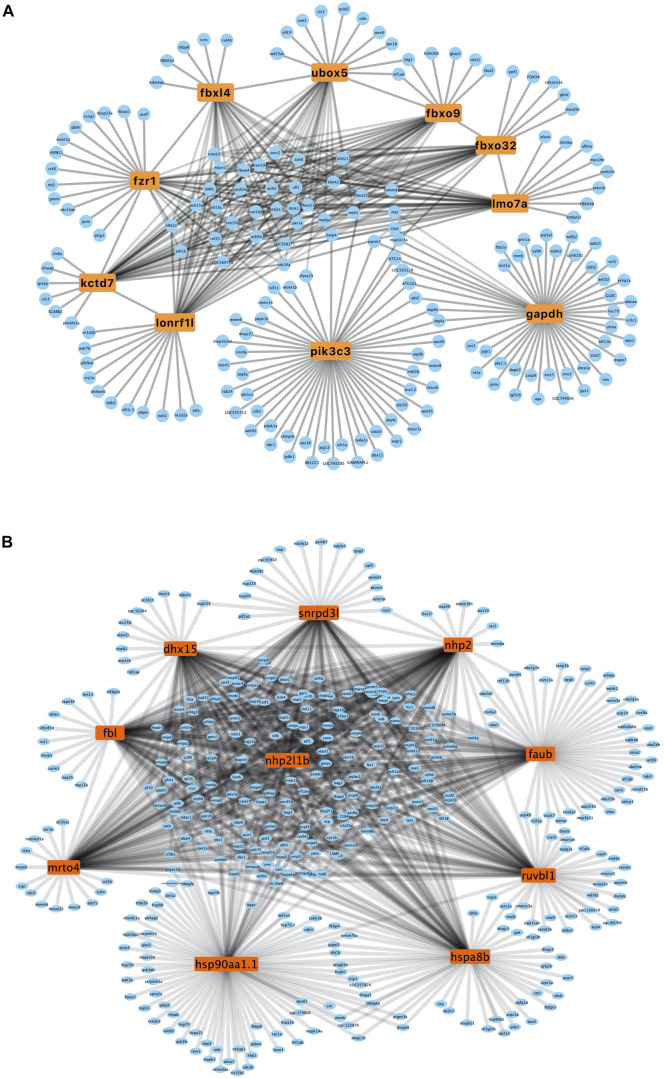
PPI network of upregulated and downregulated genes. **(A)** PPI network of upregulated hub genes. Hub genes are listed in the box. **(B)** PPI network of downregulated hub genes. Hub genes are listed in the box.

A PPI network was generated using the STRING database. The genes *pik3c3*, *gapdh*, *fbxo32*, *fzr1*, *ubox5*, *lmo7a*, *kctd7*, *fbxo9*, *lonrf1l*, and *fbxl4* were the top ten upregulated genes with the highest fold change (Table 2), and *nhp2l1b, nhp2, fbl, hsp90aa1.1, snrpd3l, dhx15, mrto4, ruvbl1, hspa8b*, and *faub* were the top 10 downregulated genes with the highest fold change (Table 2). The PPI network of these hubgenes with the interaction of DEGs was generated using Cytoscape (Figures 5A,B). We performed an additional round of GO enrichment analysis of the proteins in the PPI network. The representative 10 BP GO terms are listed in Table 3. We found that the most downregulated genes were involved in protein synthesis and modification, mitochondrial function, and sarcomere structure, while the most upregulated genes were involved in autophagy, signal transduction, and glycolytic process.

**TABLE 2:** Hub genes in the PPI networks.

	Hub gene	Description	Node
Up	pik3c3	Phosphatidylinositol 3-kinase, catalytic subunit type 3	52
	gapdh	Glyceraldehyde-3-phosphate dehydrogenase	49
	fbxo32	F-box protein 32	45
	fzr1	Fizzy/cell division cycle 20 related 1b	45
	ubox5	U-box domain containing 5	44
	lmo7a	LIM domain only 7a	44
	kctd7	BTB/POZ domain-containing protein	44
	fbxo9	F-box only protein 9	43
	lonrf1l	LON peptidase N-terminal domain and ring finger 1, like	43
	fbxl4	F-box and leucine-rich repeat protein 4	42
Down	nhp2l1b	NHP2 non-histone chromosome protein 2-like 1b	105
	nhp2	NHP2 ribonucleoprotein homolog	104
	fbl	Fibrillarin	101
	hsp90aa1.1	Heat shock protein HSP 90-alpha 1	99
	snrpd3l	Small nuclear ribonucleoprotein D3 polypeptide, like	97
	dhx15	DEAH (Asp-Glu-Ala-His) box polypeptide 15	97
	mrto4	MRT4 homolog, ribosome maturation factor	96
	ruvbl1	RuvB-like AAA ATPase 1	96
	hsap8b	Heat shock 70kDa protein 8	96
	faub	FAU ubiquitin like and ribosomal protein S30 fusion b	96

**TABLE 3:** Gene ontology enrichment analysis for the intersecting proteins of hub genes in the heart tissue of zebrafish subjected to excessive exercise.

	ID	Term	*P*-value
Up	GO:0000045	Autophagosome assembly	2.49E−14
	GO:0016567	Protein ubiquitination	4.34E−14
	GO:0000422	Mitophagy	9.89E−12
	GO:0044804	Nucleophagy	1.23E−05
	GO:0035556	Intracellular signal transduction	5.32E−05
	GO:0051697	Protein delipidation	9.85E−04
	GO:0016311	Dephosphorylation	1.02E−03
	GO:0006096	Glycolytic process	5.16E−03
	GO:0006995	Cellular response to nitrogen starvation	6.21E−03
	GO:0006289	Nucleotide-excision repair	1.86E−02
Down	GO:0006457	Protein folding	4.50E−14
	GO:0006412	Translation	8.92E−10
	GO:0006511	Ubiquitin-dependent protein catabolic process	3.25E−05
	GO:0006396	RNA processing	3.15E−05
	GO:0042254	Ribosome biogenesis	3.41E−04
	GO:0006950	Response to stress	3.57E−04
	GO:0006123	Mitochondrial electron transport, cytochrome c to oxygen	1.96E−03
	GO:0006338	Chromatin remodeling	2.27E−02
	GO:0030833	Regulation of actin filament polymerization	3.06E−02
	GO:0007017	Microtubule-based process	3.51E−02
	GO:0006886	Intracellular protein transport	7.67E−04

## Discussion

**FIGURE 6:**
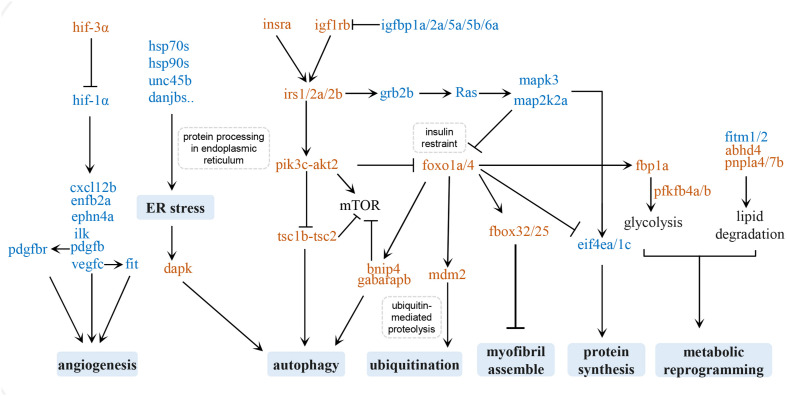
Regulatory network of exercise-induced pathological hypertrophy. Upregulated genes are in brown and downregulated genes are in blue.

Cardiac hypertrophy initially takes place as an adaptive response to hemodynamic overload to reduce ventricular wall stress. Whether it results in pathological or physiological hypertrophy depends on different underlying molecular mechanisms (Nakamura and Sadoshima, 2018). Due to the convenience of manipulation and the relatively simple genome, we have designed several swimming schemes with different durations, frequencies, and swimming speeds to train zebrafish model to study human cardiac pathological and physiological hypertrophy. Physiological cardiac hypertrophy is characterized by normal or enhanced cardiac function and normal organization of cardiac structure, whereas pathological cardiac hypertrophy is accompanied by fibrosis and cardiac dysfunction (McMullen and Jennings, 2007). In the present study, we over-trained zebrafish according to a pre-designed swimming program and found that the hearts of these zebrafish were enlarged, with increased myocardial fibrosis and disassembled myofibril and mitochondria. The hearts of zebrafish subjected to over-exercise had notable contractile impairment and cardiopulmonary function impairment, suggesting the occurrence of pathological cardiac hypertrophy. We carried out high-throughput sequencing analysis of the transcripts in pathologically hypertrophied zebrafish hearts and the hearts of a matched control group. The DEGs between the two groups were mainly involved in autophagy, protein homeostasis, myofibril assembly, angiogenesis, metabolic reprogramming, the insulin/insulin-like growth factor (IGF) signaling pathway, and the FoxO signaling pathway. These signaling pathways and factors related to these physiological changes may form a regulatory network of exercise-induced pathological hypertrophy (Figure 6).

### Insulin and the IGF Signaling Pathway

The insulin and IGF signaling pathways regulate a series of cellular processes in the heart, including cell metabolism, contractility, growth, proliferation, differentiation, and apoptosis (Saltiel and Kahn, 2001). The binding of insulin or IGF1 to the insulin receptor (insr) or IGF1R recruits and phosphorylates the adapter proteins insulin receptor substrate 1 (IRS1) and IRS2, which in turn activates the PI3K-AKT signaling pathway to preserve physiological cardiomyocyte growth (Heineke and Molkentin, 2006; Duan et al., 2010).

Although the Insulin/PI3K/AKT axis is known to promote heart function under stress by inducing physiological hypertrophy of the heart, the insulin signals may also induce pathological hypertrophy (Shimizu et al., 2010). Excessive cardiac insulin signaling or constitutive activation of the PI3K/AKT pathway could disrupt the coordination between tissue growth and angiogenesis in the heart, and cause cardiac dysfunction. In mice, PI3K/AKT upregulation after chronic pressure overload induces pathological hypertrophy of the heart (Shioi et al., 2002). Increasing clinical evidence has shown that insulin resistance, as a result of hyperinsulinemia, is the main cause of non-ischemic heart failure (Witteles and Fowler, 2008). Likewise, our KEGG analysis of DEGs in excessively exercised zebrafish hearts showed that the insulin pathway and insulin resistance were significantly enriched, and the observed unregulated expression of *insr*(*insra*), *igf1rb*, *irs*(*irs1*, *irs2b*,*irs2a*), *pi3k*(*pik3cb*, *pik3cg*), *pdk*(*pdpk1b*), and *akt*(*akt2*) may facilitate the activation of cardiac insulin signals. Interestingly, the FoxO proteins (foxo1a and foxo4) were upregulated, which may inhibit some key functions of insulin or insulin-like growth factor, including cell metabolism, autophagy, protein synthesis, and myofibril assembly (Lee and Dong, 2017; Link, 2019). These two signaling pathways may thus contribute to pathological cardiac hypotrophy.

In contrast, some of the zebrafish insulin-like growth factor binding proteins (igfbp), such as igfbp1a, igfbp2a, igfbp5a, igfbp5b, and igfbp6b, were significantly downregulated in the exercise group. The IGFBP family is a highly conserved secreted protein, and is an important regulator of the IGF signaling pathway (Firth and Baxter, 2002). These proteins mediate the signal transmission of IGFs, mainly by regulating the half-life and access of IGF-1 to its receptors on the cell membrane surface (Eivers et al., 2004; Holly and Perks, 2006). Increased IGF1 levels in serum are associated with physiological cardiac hypotrophy in athletes (Neri Serneri et al., 2001). IGFBP2 plays an essential role in maintaining physiological hypertrophy (Olszanecka et al., 2017). The increased synthesis of IGFBP2 may be related to a protective mechanism against excessive cardiac remodeling (Rehfeldt et al., 2010; Sharples et al., 2013). Low levels of IGFBP2 are independently associated with a lower stroke volume index, which is a powerful predictor of poor prognosis in patients with aortic stenosis (Carter et al., 2015). Low stroke volume in patients with aortic stenosis is a typical symptom of left ventricular concentric remodeling, which initially compensates for increased cardiac pressure load, but then leads to fibrosis and heart failure. In the present study, significant downregulation of igfbp2a, igfbp1a, igfbp5a, igfbp5b, and igfbp6b was detected, which may be one of the possible reasons for cardiac fibrosis and heart failure in excessive exercise-induced pathological hypotrophy.

In general, insulin and IGF signals are associated with physiological cardiomyocyte growth. In this excessive exercise-induced pathological hypotrophy model, the activation of cardiac insulin and IGF signaling is also accompanied by the upregulation of the FoxO signaling pathway and may be a cause of cardiac pathological remodeling.

### Protein Homeostasis

We identified several key protein synthesis pathways associated with DEGs in the hearts of excessively exercised zebrafish, suggesting a reduction in the capacity to produce functional proteins. The expression of spliceosome components, such as *snrpd1*, *snrpd2*, and lsm6, was downregulated, which has the effect of decreasing the initiation of synthesis of proteins in cardiomyocytes. We also identified the downregulation of proteins involved in protein endoplasmic reticulum processing, especially hsp70s (hsp70.1/hspa8b) and hsp90s (hsp90aa1.1/2), and their co-chaperones (Figure 3), which may lead to aberrant protein folding (Hartl et al., 2011). The accumulation of misfolded proteins can cause endoplasmic reticulum pressure, promote the expression of dapk protein, and induce protein ubiquitin degradation and autophagy. Correspondingly, the proteins involved in autophagosome assembly, mitophagy, autophagy, and protein localization to pre-autophagosomal structures were upregulated after excessive exercise. The upregulation of autophagy induction complex-tcs1/tcs2 and “core” autophagy machinery, such as the ulk1 complex (ulk1a, ulk2, atg13, atg101, and rb1cc1), pik3c3 complex (atg14 and pik3c3), atg8 conjugation system (map1l3c3a, map1l3c3cl, atg4c, and gabarapb), and atg9 complex (atg9a, atg9b, wipi1, and wdr45) may provide a protective mechanism for protein folding disorders to maintain proteostasis (Mathai et al., 2017). Increased mitophagy is an indicator of mitochondrial damage and dysfunction, and is one of the typical features of pathological hypotrophy (Nakamura and Sadoshima, 2018).

### Altered Sarcomere Structure and the Blockade of Myofibril Development

The expression of the effectors of the FoxO pathway, such as *foxo1a*, *foxo4*, *fbox32*, and *fbox25*, was upregulated in the hearts of excessively exercised zebrafish, which may lead to myopathy (Shimizu et al., 2017). Pathological cardiac hypertrophy is the most common primary cardiomyopathy, which represents a disease with decreased myocardial contractility and insufficient cardiac pump function. The cause of many cardiomyopathies is the structural abnormality of the cardiac myofibrils (Brieler et al., 2017; Dadson et al., 2017; Lin et al., 2019).

According to enrichment analysis, myofibril development and sarcomere organization were affected in zebrafish hypertrophic hearts. The downregulation of related genes, such as *tnnt3b*, *myoz1a*, *klhl41a/b*, *hsp90aa1.1*, and *unc45b*, influences the assembly, repair, and contraction of myofibrils. Mutants for klhl41 and klhl40 have disrupted muscle structure and loss of movement (Gupta et al., 2013; Ravenscroft et al., 2013). Likewise, unc45b and hap90aa1.1 play an essential role in the assembly of thick filaments of sarcomeres, and the functional deletion of unc45b resulted in a complete loss of motility and disorder of myofibrils in zebrafish hearts (Etard et al., 2007; Hawkins et al., 2008; Myhre et al., 2014). To restore the homeostasis of myofibril after damage or under stress, hsp90aa1.1 and unc45b dissociate from the Z line and translocate to the A bands of the myofibril to repair myofibrillar damage and restore sarcomere assembly (Etard et al., 2008). Troponin is the constituent protein of sarcomeres and is essential for the regulation of Ca^2+^-dependent contraction. In humans, a variety of cardiomyopathies are associated with mutations in all three cardiac troponin subunits (TNNT1, TNNT2, and TNNT3). The depletion of zebrafish tnnt3b, the ortholog of human TNNT3, causes deficiency of troponin activity and sarcomere disintegration, probably due to deregulation of actin-myosin activity (Ferrante et al., 2011).

In addition to the genes affecting sarcomere organization, T-tubule organization-related genes, such as *tcap*, *dag1*, *dysf*, and *cavin4b*, were also downregulated. The sarcolemma penetrates into the intracellular space of myofilaments to form cardiac T-tubules, which are rich in concentrating voltage-gate L-type calcium channels and signal transduction molecules, and plays a central role in myofibril electrophysiology and sarcomere contraction. Zebrafish *dag1*, *tcap*, or *cavin4b* mutants display T-tubule abnormalities and progressive muscle dysfunction (Zhang et al., 2009; Gupta et al., 2011; Housley et al., 2016). The *dysf* zebrafish morphants also showed misaligned and fragmented T-tubules. Ordered protein-membrane scaffold assembly for sarcolemma repair begins with the formation of the dysf and anxa6 complex at the lesion (Roostalu and Strahle, 2012).

Adult zebrafish cardiomyocytes possess the ability to regenerate, which permits them to restore the heart after substantial amounts of damage, while the inhibition of myofibril development and sarcomere assembly may block the regeneration process. Excessive exercise causes the downregulation of genes that enable regeneration, and therefore, the injured heart is unable to recover to homeostasis, eventually resulting in apoptosis or necrosis.

### Insufficient Angiogenesis

In response to an increase in pump pressure, the myocytes and capillary microvasculature grow harmoniously to increase heart weight, resulting in enlargement (Oka et al., 2014). However, the disproportional growth of myocytes and capillaries leads to insufficient capillary density and coronary flow for oxygen and nutrient supply, resulting in myocardial ischemia in pathologically hypertrophied hearts (Nakamura and Sadoshima, 2018).

Hypoxia-inducible factors (HIFs), which are important transcription factors in response to hypoxia, play an important regulatory role in the angiogenesis of pressure-overloaded hearts. HIFs are heterodimers composed of an unstable α-subunit (HIF-1α, HIF-2α, and HIF-3α) and a stable β-subunit (HIFβ) (Sousa Fialho et al., 2019). The downregulation of HIF1α expression or the inhibition of HIF1α function can reduce angiogenesis and cause maladaptive hypertrophy during chronic pressure overload. We found that the expression of hif1α (hif1ab) was downregulated in excessively exercised zebrafish hearts, while the upregulation of hif3α might competitively combine with hifβ (hif1al), which further weakens the function of hif1α (Zhang et al., 2014). At the same time, angiogenesis factors related to hif1α, including pdgfb, vegfc, cxcl12b, efnb2a, ephn4a, ilk, and fit1 (Semenza, 2014), were also significantly downregulated in the exercised zebrafish, suggesting that angiogenesis was inhibited in our excessive exercise-induced hypertrophic model.

### Metabolic Reprogramming

Although appropriate metabolic adaptation to pathological hypertrophy may produce harmful intermediate metabolites, cardiomyocytes still undergo metabolic reprogramming to maintain contractile function.

Fructose, mannose, and other glycogen degradation increased during excessive exercise, and the glycolysis rate-limiting enzymes, such as fbp1a and pfkfb4a/b, were significantly upregulated, suggesting that carbohydrate metabolism becomes essential under these conditions (Strohecker et al., 2015). Another significant metabolic change was related to lipid metabolism. The observed downregulation of fitm1/2 results in a decrease in the storage of triglycerides, the most important source of stored energy in cells, in lipid droplets (Gross et al., 2010). Concurrently the enzymes that decompose triglycerides and phospholipids into fatty acids, such as lipia, gba, pnpla7b, pnpla4, abhd4, prxl2, pla2g15, and asah1a (Schaloske and Dennis, 2006; Gao et al., 2009; Lelieveld et al., 2019), were upregulated, suggesting that the lipids were broken down into fatty acids to provide energy during excessive exercise in zebrafish.

We also observed the downregulation of enzymes related to terpenoid backbone biosynthesis, such as pass1, pdss2, idil, acat2, and ggps1. Terpenoid backbone biosynthesis involves the use of acetyl-CoA to synthesize physiologically active substances (Mendoza-Poudereux et al., 2015). As a metabolite of glycolysis and fatty acid oxidation, acetyl-CoA is mainly used to enter the tricarboxylic acid (TCA) cycle to produce ATP, perhaps owing to the inhibition of terpenoid backbone biosynthesis under the pressure of energy consumption. Interestingly, ubiquinone is the product of terpenoid backbone biosynthesis and is also an important component of the mitochondrial respiratory chain. Thus, disruption of terpenoid backbone biosynthesis may possibly contribute to mitochondrial dysfunction and pathological cardiac remodeling.

## Data Availability Statement

The datasets generated for this study can be found in the NCBI Sequence Read Archie (SRA) accession PRJNA635689.

## Ethics Statement

The animal study was reviewed and approved by the Ethics Committee of Hunan Normal University (approval number: 2018/046).

## Author Contributions

XP, PZ, and XW designed the experiments. ZC and RZ collected the zebrafish sample. ZZ, XP, and LZ performed the experiments. ZZ, XP, LZ, and CT performed the analysis of the experimental results and RNA-seq. ZZ, XP, XW, and PZ wrote the manuscript. All authors have read and approved the final manuscript.

## Conflict of Interest

The authors declare that the research was conducted in the absence of any commercial or financial relationships that could be construed as a potential conflict of interest.

## Footnotes

1 http://eggnog5.embl.de/#/app/home

2 https://string-db.org/

3 https://cytoscape.org
