# Supplementary material for: Identification of Potentially Relevant Genes for Excessive Exercise-Induced Pathological Cardiac Hypertrophy in Zebrafish
Source: Front Physiol. 2020 Nov 30;11:565307. doi: 10.3389/fphys.2020.565307 (PMC7734032; doi:10.3389/fphys.2020.565307)
Supplement: Supplementary file 5 [file Data_Sheet_1.DOCX]

**Supplementary Materials**


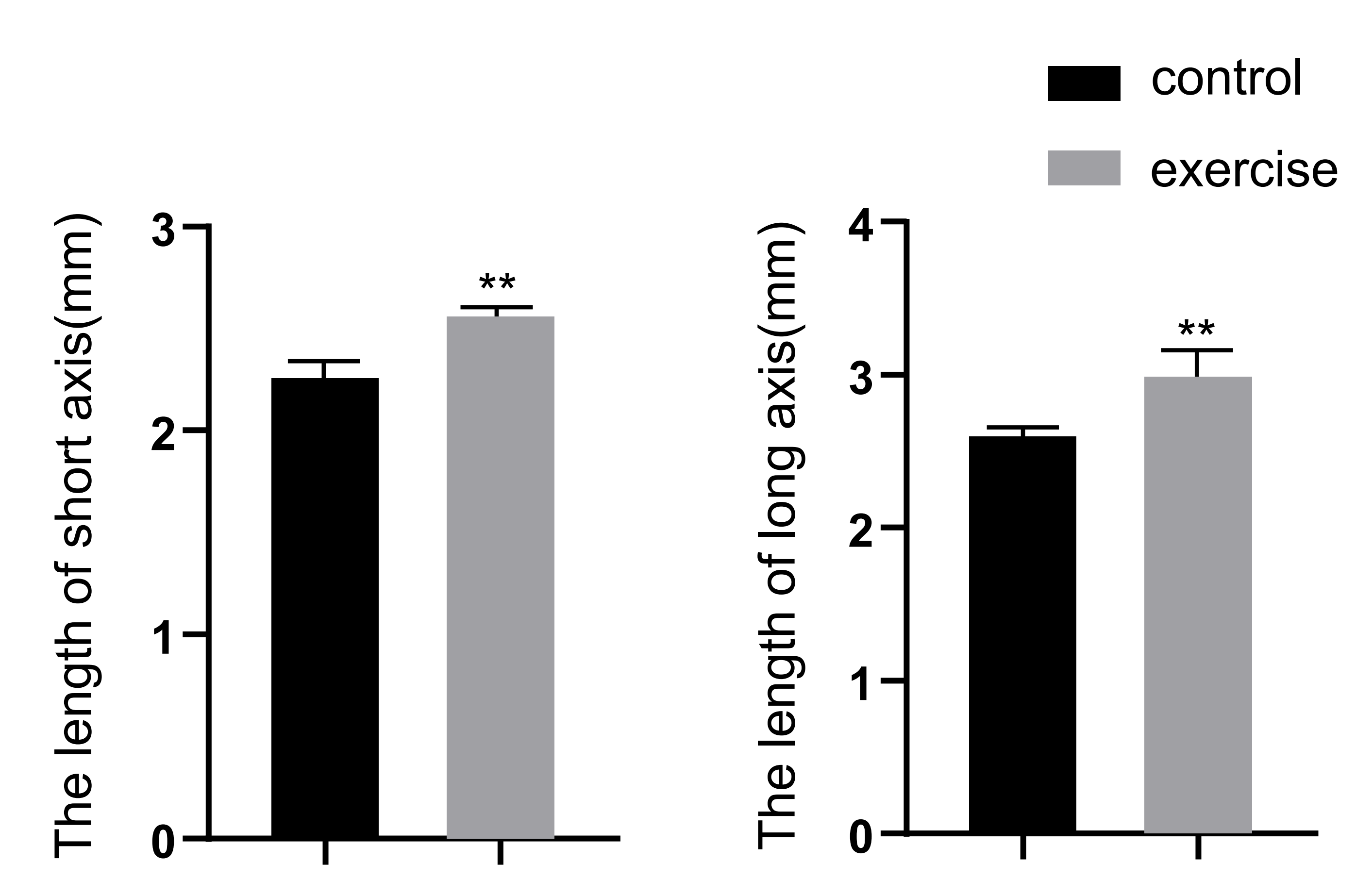


**Supplementary Figure S1.** The length of short axis and long axis in excessively exercised group were both longer than the heart size measurement. n=8 in each group. **p<0.01 by unpaired Student's t-test.

**Supplementary Table1: Primers for RT-qPCR**

| *Gene* | Forward primer (5'-3') | Reverse primer (5'-3') |
| --- | --- | --- |
| *atg101* | ACGTGGACTGCGACTTCATA | GAATTGCCAAGCGCATCCTTAAA |
| *atg9a* | ATGACATCCTCTTCGCCAACAA | ACACTGTCACGGATCCTAGC |
| *atg9b* | TTCACTACATGCCCGACCAC | CCTCCAAGATGAAGACCGCT |
| *bnip4* | AGCGGAACAGAATATGCAACAC | CCGTCCAATACAGATCCCCAATC |
| *dapk* | GACGACAATGTTCCCGGTCT | CGGAGGAGTTCCATGCTTGT |
| *cox4i1* | GGCAACTACGGCATTTCGTC | CGACCTTCGCAACTCCATGT |
| *cyc1* | TGTGGTCGTGTTCTCCGGTA | AATGACATACTGGCCCGTGG |
| *fbox32* | CACAGACAGACAGATCCGCAA | GCAGTGCAAGGATGGTCTGT |
| *foxo1a* | AGCGGCAAAGAAAAAGCTGG | CCGTCCAGGCTTCAAAGTCA |
| *foxo4* | CAGGGAATGCAGACAGTCCT | AGCCACCAAGAAGTCCATCC |
| *hif3a* | TGGCTGCTAGATGGGACAAG | CCATCAGACAAGCCATCCAGT |
| *insra* | GAACTATACAGTGCGGATCAG | TGGACCAATCACAATCTTCAG |
| *irs2a* | CGCCTGATGATGGGACTTGT | GAACCCTGTTAGTGGGGCTG |
| *irs1* | ACTCCACTTCCGGACGAAAC | TGACAGGTGCTCGATGTGTC |
| *pfkfb4a* | ACTGTAACACAGAAGAGGCCG | GAAGGAAAGATCCCGGTCCAG |
| *pik3c3* | CCTGGCCAAGCTAACAAAAGC | GAGCTACGCTTCTCACTCTCA |
| *tsc1b* | AGCTTACTGCCAAGTTGTCCT | TATCCGGTCCAGCCTGATGA |
| *tsc2* | GTTTGACGACACACACGAGC | TTGGTCGTCCGCAAACTCTT |
| *ubox5* | TCCACACGCACACAGAGTC | GTAAGCCATCAAGTGCAGCG |
| *wdr45* | GCAGTCTGCAGTTAGTGGATCT | CGCTGCCCGGCTGATT |
| *cavin4b* | GTCAGCTGCCACATCAAGGA | GGGACCTCATTATCTCCCTGGTA |
| *dag1* | CCGGGAGCATAGTAAAGATCACA | TGTAGTGAACGCCCTTGTCC |
| *dhx15* | CCGCTATGCTATCAGTCCCAC | CAGCGAGCGGTAGTTGATGA |
| *faub* | GGTCAGAGACTGTTGCCCAT | GAACCGTGAACTTTGCCTCCT |
| *fitm1* | CGCATCCTCTTCCTGTTCTG | AAACAACCCAAAGCACCTCC |
| *fitm2* | CAGGTTCGTGCTATGAGTCCA | CCATCCCAGAAGAAGCCAGC |
| *hsp70.1* | ACTTACTCCGACAACCAGCC | AAGGTCACTTCGATCTGCGG |
| *hsp90aa1.1* | CAAACTCGGACTTGGCATCG | CCCTCCAGAACGGGCATATC |
| *hsp90aa1.2* | TCAAACTGGGCTTAGGCATTGA | CTCCTCTATCGGGGCAGAAC |
| *hspa8b* | CGTCACCCCTCTGTCTTTGG | CTCCCCCTCGTACACCTGAAT |
| *igf1rb* | TGCCAGACTACGACCGATTC | ACATGCTGACAGACACACCAT |
| *igfbp1a* | TCGAACTCCAGACAGCCCTT | CCATCTAGAGATGATTCGCACTGTT |
| *igfbp2a* | GGCAGTACAACCTGAAACAGTGTA | TATCAGTGGAGAGGTGGGCA |
| *igfbp5a* | GCGAAAGGATCGGAAGAGACA | TTCGGCATGGACCAATGTCAG |
| *lsm6* | CTGGTGTCGATTACAGAGGAGT | TTGTTCTTCAGTTGGCCGTTG |
| *map2k2a* | ACGACCAGTTAGTGGACACG | TTGGGTGGAGGCTCATTGAC |
| *mapk3* | ATGCTTAACTCCAAGGGCTACAC | GCTGGTCCAGATAGTGCTTCC |
| *mrto4* | AACTTGATTGAAGAGCTGCGGA | TCCTTTACCCAGCGCAATCAT |
| *myoz1a* | ACGAGCTATGAAGGGCAACG | AAGGCATGGCACTCCTGTTG |
| *nhp2* | GCGTCAAGAAAGCGGCTAAA | TACGGCAGGCTTCTGTCTTC |
| *nhp2l1b* | CCCTAAAGCCTATCCTCTGGC | CGGTTAAGAGTTTTGGTGGCTTC |
| *pdgfb* | CAAGACCCTGCAGTGTGTTC | CACGTGCGTCTAGTTTCTGG |
| *snrpd1* | GGCACAATTACAGGTGTGGAC | TCCGGCAGGATAAAGTAGCG |
| *snrpd2* | GTGCTCACCCAGTCTGTGAA | AGAACCATATTACAATGCCTGTCG |
| *snrpd3l* | AACATGAACTGCCAGATGTCCA | CACGGATGTAGACCTGCTCG |
| *tcap* | CAACCTGAGGATCGCCAGTC | CTGGTGCTCTCTTACACCCG |
| *tnnt3b* | GTACTCCCTGGAGGCTGAGA | GGCGCCCTTCTTGGAGTATT |
| *vegfc* | AAGCAGATGCCATGCAGGAG | CATCCACACTACCCGCTGAA |
| *nd1* | AGCCATCTCAAGCCTAGCAG | ATTGTTTGCGCTACAGCTCG |
| *nd2* | GACCTACCAGCCACAGCTAC | TTGGGTCGTTTGTACCCGTC |
| *nd3* | ACCACTCCCATGAGGAGATCA | CTTGGGCTCATTCGTAGGCT |
| *nd4* | CCAACCTAGCACTTCCACCT | TCTGGGATTGAGCCTCGTTG |
| *nd5* | TCGCCACATCATTTACCGCA | TCAGGCAAGCCGTTGAATAGT |
| *nd6* | CCGCCACAATTACAACCAGAC | TTTGCTTATTCAGCGGCCCTA |
| *vdac1* | TTAACCTGGGCTGTGACGTG | ACAGCGAAGTTGCTCTGTGT |
| *pik3r1* | TGCCAGACGCAAGAACGATA | GCTTAATGCTGTTCAT CCGCT |
| *eif4ea* | CCATC GTACACATAGGGCGT | TGTCCGCATGTGACTGGTAT |
| *nppa* | GACTGCTGCTCCTGGTTTG | CTGTCTTCATAATCTACGGCTC |
| *nppb* | AGCAAACT GGACAGAATCGGT | TAAATCAAGTAGAAACAACGCAT |
| *myh7* | TGGTGAGGGAGGAAA GAGCAT | CGCAGAATCTTACCCTCCTCG |
| *actc1* | GACCCACAACGTGCCCATCT | AATCTCACGCTCGGCAGTGG |
